# A Practical Approach to Artificial Intelligence in Plastic Surgery

**DOI:** 10.1093/asjof/ojaa001

**Published:** 2020-01-08

**Authors:** Akash Chandawarkar, Christian Chartier, Jonathan Kanevsky, Phaedra E Cress

## Abstract

Understanding the intersection of technology and plastic surgery has been and will be essential to positioning plastic surgeons at the forefront of surgical innovation. This account of the current and future applications of artificial intelligence (AI) in reconstructive and aesthetic surgery introduces us to the subset of issues amenable to support from this technology. It equips plastic surgeons with the knowledge to navigate technical conversations with peers, trainees, patients, and technical partners for collaboration and to usher in a new era of technology in plastic surgery. From the mathematical basis of AI to its commercially viable applications, topics introduced herein constitute a framework for design and execution of quantitative studies that will better outcomes and benefit patients. Finally, adherence to the principles of quality data collection will leverage and amplify plastic surgeons’ creativity and undoubtedly drive the field forward.

“AI is not a doctor, AI is not a cure, AI is a tool – a means to an end – increasingly embedded in everything for the benefit of the patient”—Keith Bigelow, General Manager, GE Healthcare.

“The computer scientist Donald Knuth was struck that ‘AI has by now succeeded in doing essentially everything that requires ‘thinking’ but has failed to do most of what people and animals do ‘without thinking’—that, somehow, is much harder!’”—Nick Bostrom, Superintelligence: Paths, Dangers, Strategies.

Current interest in artificial intelligence (AI) is unequivocal—we are fascinated as healthcare professionals, but more generally as curious members of humankind. For most of us, however, our exposure has been extremely limited. As the technology community romanticizes AI and packages it into commercial products for resale to the healthcare industry (and all other industries for that matter), we on the front lines cannot help but wonder how to stay ahead of the curve. How might a research team grappling with study design and difficult data collection best position itself to leverage open-source AI resources in the name of efficiency and better patient outcomes? How might the individual surgeon use AI tools to assist in optimizing surgical plans or improving the patient experience? This account should provide plastic surgeons with a practical approach to understanding the uses, limitations, and potential of artificial intelligence in research and clinical practice.

## WHAT IS AN AI PROBLEM?

An introduction to the various applications of artificial intelligence is best driven by the following question: What is—and what is not—an “AI problem?” Identifying areas amenable to support from artificial intelligence should be the first step towards AI fluency.

It is important to first understand AI as a blanket term used to describe pattern recognition across massive data sets. Amazon leverages the features of commoditized transactions involving millions of customers and products to predict the best product you have not yet thought about buying. Specifically, AI is only at play here to the extent that a powerful computer can (1) scan a large database to identify peers (clusters of customers with similar purchasing trends) and (2) draw your attention to products you have not yet bought, but that your cluster has demonstrated interest in. Different servers, algorithms, and data set sizes do this with varying degrees of success. From this perspective, the broad features of AI problems are much easier to elucidate. Google, Spotify, and Netflix all use similar approaches to modeling human preference and predicting user actions.^1^ Other fields apply this same strategy in less intuitive ways: credit card fraud and email spam filtering are both glorified pattern recognition problems.^2,3^

No matter how pervasive this technology has become, one must not stray from the basic understanding of AI as pattern recognition. The quintessential example is that of an AI algorithm trained to differentiate cats from dogs through what is called image recognition. Preliminarily, when shown an image and asked to classify it as either a cat or a dog, an algorithm is no more or less likely to choose “cat” or “dog” than would be a human flipping a coin. Gradually, however, as the algorithm sees an increasing number of images, it can be fine-tuned to begin correctly differentiating cats from dogs. Having been fine-tuned and after looking at thousands or millions of images, the AI is now capable of human-level animal recognition simply by having learned the pattern.

In medicine, artificial intelligence has been well-received by the radiology community because radiologists interface with a large quantity of standardized data. Plain films are more frequently the subject of studies relating to AI than are magnetic resonance imaging (MRI) studies because they are subject to less interoperator variability. A chest x-ray from one hospital is likely to be more similar to a chest x-ray taken at another hospital than are two MRI studies performed in different centers.^4^ Standardized advanced imaging is now used by technology platforms such as Crisalix (Crisalix, Switzerland) and Vectra (Canfield Scientific, Parsippany, NJ) to simulate surgeries (breast, head, and neck) in the preoperative setting. It follows logically that areas of medicine that are similarly reliant on high-quality standardized data are increasingly studied by data scientists. For example, the MIT Lab for Computational Physiology developed MIMIC, a freely accessible critical care database comprised of demographics, vital sign data, laboratory test results, procedures, medications, medical notes, imaging, and mortality for more than 40,000 critical care patients between 2001 and 2012.^5^ Countless seminal studies at the intersection of healthcare and AI have relied on the MIMIC database and others like it.^6,7^ Successful uses of AI in other fields of medicine, such as diagnostic ophthalmology, dermatology, precision medicine, and pathology, have also been reported on.^8–11^

Unsurprisingly, given the precondition of plentiful standardized data, artificial intelligence has had few successful use-cases in surgical fields. In surgery, thought leaders hobble towards consensus, publishing their preferences in case series with limited enrollees, suggesting sparsely available data and high interoperator technical variability, which has been a limitation to the use of AI in plastic surgery. Thus, we propose two approaches to the integration of AI in plastic surgery: higher-quality data collection and feature engineering.

## HIGHER-QUALITY DATA COLLECTION

Data collection has historically been the bane of surgical teams. Surgeons are overworked, their craft is time-sensitive, and complications have dire consequences on patient wellbeing. These features of surgery make the integration of technology a war of attrition. Technologists, whose clever products may be backed by strong data, often fail to get their proverbial foot in the operating room door because they are insensitive to the “ergonomics” of being a surgeon. If they fail to sell to surgeons as an extremely subspecialized category of customer, their products will be viewed as cumbersome and adoption will be slow and painful. The same pain points are relevant to surgical data collection.^12,13^ Countless clever projects have failed at standardizing operating rooms for the purpose of collecting high-quality surgical data because seamless integration was not made a priority.

AI enthusiasts are more likely to have their data collection strategy widely adopted by being mindful of clinic and operating room workflow. The current standard of quality OR data collection is through the use of video.^14^ Namely, Hashimoto et al described their recording of laparoscopic sleeve gastrectomies for quantitative video analysis by algorithms.^15^ With a greater emphasis on standardized data collection, prospective studies can be designed for eventual algorithmic analysis.

In plastic surgery, repositories of preoperative and postoperative two- or three-dimensional imaging have great potential to harness the powers of AI. However, standardization of these images, including angles, lighting, expressions, hair/makeup, not only is difficult to achieve between different surgeons, but often is poorly standardized within the same patient! As we push towards advocating for photography standardization to enable peers and patients to properly evaluate results of new techniques and technologies, the missed opportunities with AI should also be used to bolster the efforts.

## MORE CREATIVE USE OF CURRENTLY AVAILABLE DATA—FEATURE ENGINEERING

Although Hashimoto et al successfully collected surgical footage for the purpose of analysis by algorithms, data collection remains resource-intensive. This has given rise to what data scientists call “feature engineering.” Feature engineering refers to the process by which experts “augment,” or annotate data to help achieve functional algorithms with less data. The classic example of feature engineering in healthcare AI is the annotation of histological slides. Healthcare startup PathAI is the leader in AI-assisted pathology laboratory interpretation, relying on trained pathologists to annotate clinical slides and achieving greater diagnostic accuracy than any human pathologist.^16^ By employing expert interpretation to data before introducing it to the algorithm, researchers can decrease the amount of data required to reach a meaningful conclusion.

To establish which of the above strategies will help most effectively integrate AI into your specific plastic surgery research project or practice, it is important to gain a more granular understanding of the mathematical principles behind AI.

## AI MATH

Part of the luster of artificial intelligence lies in its complexity. In reality, the degree of complexity of most of the peer-reviewed literature on AI is bimodal. Authors either delve into great theoretical detail on their algorithm of choice, or they spend almost no time at all describing the technical aspects of their methodology.^17^ The authors have found the following conceptual understanding of “AI math” to be sufficient to orient oneself for the purposes of AI-related plastic surgery applications:

### What Is an AI Algorithm?

In artificial intelligence, the term algorithm describes the mathematical relationships between input data and output predictions—with the basic understanding being that a fully functional (“trained”) AI algorithm can make predictions based on a set of feature-inputs. Examples of validated AI algorithms and models include decision trees, naïve Bayes, k-nearest neighbors, and support vector machines.^18–21^

### What Is an AI Data set? What Are Features? What Are Training and Test Sets?

In artificial intelligence, a data set can be thought of as a table of *y* instances of *x* features. For example, a database of antibiotic resistance profiles (15 antibiotics) on 100,000 patients may be described as having 100,000 instances of 15 features. The linear algebra term “dimension” is often used instead of “feature.” When higher-order data are involved (images and video), the number of features can increase dramatically, with a corresponding increase in the number of instances required to reach a meaningful conclusion. Typically, data scientists expect a minimum of 5 instances per feature when training an algorithm.

To train an algorithm is to teach it to perform a specific task. Using the example above, an algorithm may be trained using data on resistance to 14 antibiotics to predict resistance to the 15th. Training an algorithm for this task requires segmenting the available data (100,000 instances) into training and test sets. The training set is first shown to the algorithm with the prediction variable (the “label,” positive or negative resistance to the 15th antibiotic in this case) visible. This is akin to a student studying a problem set and answer key before an exam. Once trained, the algorithm is shown the remaining instances (the test set) with labels hidden and asked to make predictions about resistance to the 15th antibiotic. This is akin to a student taking a similar test without access to the answer key. Training-test splits vary but often divide data sets into 70% training instances and 30% test instances.

The overarching goal of training and testing is “generalizability.” To successfully label test set instances, an algorithm must not simply memorize a training set. It must organically learn to recognize features of the training set that are transferable to the test set. In AI, benign memorization of training set features with limited success on a test set is called “overfitting.”

### How Does an Algorithm Learn? What Are Weight Updates? What Are Loss and Optimization Functions?

Like a new pet, algorithms learn by being discouraged from being incorrect and rewarded for being correct, such as described in Skinner’s theory of operant conditioning. In AI, these events are described in real numbers through implementation of a loss function, a quantification of how incorrect the prediction is. A familiar and simple example of use of a loss function from grade school statistics is generation of a regression line to a set of data points. Similarly, as an algorithm is fed a training set and prompted to make a prediction, a loss function quantifies and sums the degree to which the algorithm was incorrect on each individual training set instance. Examples of popular loss functions include the mean square error, mean absolute error, and mean bias error functions.

The un-tuned preliminary algorithm has now seen each instance of the training set once (known as the first epoch) and the loss function has quantified its sum inaccuracy. Logically, the aim is to fine-tune the algorithm to minimize the loss function, which would correspond to the conditions under which the algorithm reaches maximum predictive accuracy. Fine-tuning requires the use of weight updates and an optimization function.

During the first epoch, feature weights (the degree to which each feature in an instance is considered when making a prediction) are initialized randomly for the sake of obtaining a first loss function output. Feature weights are then adjusted according to a learning rate such as to progress slowly towards the global minimum of the loss function. The learning rate is simply the upper-bound magnitude by which feature weights can be adjusted from one epoch to another. Different schools of thought exist on the optimal learning rate to use—with relatively large learning rates converging towards the global loss minimum in few epochs but failing to actually reach it and small learning rates actually reaching the global minimum but in many more epochs. The most basic protocol for progressing from initialization to the global minimum on the loss function is known as gradient descent.^22^

Once feature weights have been adjusted and the global loss minimum has been reached, the algorithm is ready to be tested on the test set. Sensitivity and specificity are among many standard metrics used to determine an algorithm’s predictive accuracy on a test set.

While the researcher or clinician may never actually design or create these algorithms independently, knowledge of the basic tenants and terminology will help the dialogue with your data scientist and assist in understanding the needs and limitations of your “ask.”

## CURRENT SUB-FIELDS OF HEALTHCARE AI

Now that the basic intuition required to understand artificial intelligence has been established, it is useful to segment current research into three sub-fields of AI: machine learning and neural networks, computer vision, and natural language processing (NLP).

Machine learning and neural networks constitute the bread and butter of AI research as described above. Many layers of complexity can be added to this brand of project, though the intuition remains the same: using gradient descent to minimize a given algorithm’s loss function. Examples of projects in this category include risk modeling of complications in diabetes, optimizing heart disease diagnosis, and disease diagnosis based mainly on blood serum measurements.^23,24^

Computer vision is a mainstay of multimedia analysis and classification. Problems in this category range from basic image classification—differentiating between cats and dogs—to self-driving cars. Computer vision underpins the video segmentation work by Hashimoto et al described above and will likely eventually be the basis for surgeon-less robot surgery.^25^

The NLP technology is used to help computers interpret human speech and language. From a first principles perspective, NLP problems can be thought of as high-order machine learning problems, with massive amounts of text or recorded speech required to understand and simulate human dialogue. In healthcare, NLP specialists are currently focusing on the interpretation of text contained in electronic medical records to lean out the chart-keeping process.^26^ Other applications of NLP include hospital interpretation/translation, healthcare-specific speech-to-text dictation, and the automation of administrative tasks using chatbots.

## CURRENT STATE AND PATH FORWARD FOR AI IN PLASTIC SURGERY

Although data collection remains a challenge in all fields, plastic surgery and aesthetic surgery—insofar as they are innately visual specialties—are uniquely suited to embrace artificial intelligence. Before and after photos are widely publicized and are a testament to the success or failure of a plastic surgery procedure, intimately linking visual features to outcomes. Progress made in computer vision and facial recognition outside of healthcare is being leveraged to segment facial anatomy, quantify visual appeal, model procedure outcomes, and predict aging.^27–29^

Currently, published applications of artificial intelligence in plastic surgery are limited to the analysis of radiologic studies and information contained in medical records, examples of which have been published by de Brito et al and Choi et al.^30,31^ No consensus applications relating to plastic surgery, surgical technique, or nonradiologic diagnostic approaches exist in the literature.

As access to data increases, AI will become intricately woven to the pre-, peri-, and postoperative arenas, with patient-specific features guiding procedure selection, intraoperative decision-making, and early detection of complications. Specifically, AI will aid with diagnostic accuracy, preoperative virtual planning, disease progression, and postoperative monitoring.

Low-hanging fruits and current uses of this technology include soft tissue deformation prediction, data-driven treatment of peripheral nerve injuries using automated neuroprostheses, early detection and the planned correction of congenital craniofacial abnormalities (using both imaging and genetic studies), and the objective assessment of rhinoplasty and facial rejuvenation procedures.^32–36^ AI algorithms are also being used to assess wound depth, surface area and perfusion, with similar principles being applied to flap-based reconstruction procedures.^37^

Artificial intelligence as used in plastic surgery will also be patient- and client-facing. Data-driven surgical simulation applications capable of identifying objective asymmetries in preoperative images will provide guidance on the most appropriate method of achieving a desired cosmetic outcome. Currently, photo editing applications boasting these features seed unrealistic expectations and fail to account for the limitations of the realities of aesthetic surgery. This conservative approach to aesthetic surgical planning is economically and medically sounder and will quickly outcompete legacy procedure selection schemes. It will also spawn the practice of “prophylactic aesthetics” whereby surgeons can market preventative procedures, though an analysis of the underlying ethical framework is beyond the scope of this article. From a surgical screening perspective, AI applications are being developed to identify patients for whom specific surgeries are too risky and ruling them out in the preoperative setting. These rely on unintuitive risk factors hidden in data on previous surgical complications and have huge health outcomes and economic upsides. In the current environment of breast implant-associated anaplastic large cell lymphoma (BIA-ALCL), these applications are likely to gain significant traction.^38,39^

Furthermore, the plastic surgery community has recently demonstrated its willingness to embrace big data through development of the Tracking Operations and Outcomes in plastic surgery (TOPS), General Registry of Autologous Fat Transfer (GRAFT), National Surgical Quality Improvement Program (NSQIP), CosmetAssure, and the ASAPS.CLOUD databases. These represent a commitment to standardized plastic surgery data collection and an open-mindedness to disruption by artificial intelligence. Lastly, the Aesthetic Neural Network (ANN), launched by the American Society for Aesthetic Plastic Surgery (ASAPS), is an early-stage tool designed for practice optimization and economic modeling. These tools and databases promise to become essential features of a competitive plastic surgery practice in the near future.

There are two clear limitations to the adoption of AI in plastic surgery. Sharing of patient data is an ethically and bureaucratically challenging process in all fields of medicine. Open-source datasets are uncommon—and extremely valuable—because anonymizing data and gaining authorization for it to be made freely available to other data scientists is uncharted waters in medicine. This has resulted in researchers relying on fragmented datasets siloed from other teams and unable to benefit from the scale of data available in other fields. Specifically, in plastic surgery, collecting standardized data is an extremely resource-intensive process given increased fragmentation, especially in private practice. Current workflows are operator-dependent and tailored to individual surgeons and research projects. This is not conducive to generating the kind of data sets that can be leveraged by artificial intelligence algorithms. As described elsewhere in the text, lack of quality data harms the predictive accuracy of any validated algorithm. This manuscript hopefully serves as a call-to-action to improve sharing of data and creating standardized data sets for artificial intelligence analysis.

## CONCLUSION

Although there is an increasing appetite in the plastic surgery community for expert peer-reviewed literature on the applications of artificial intelligence in surgery, it is important to note that the democratization of access to AI resources currently makes it possible for plastic surgery innovation to come from within our community. Surgical skills and clinical intuition are necessary but insufficient conditions to implementing such powerful technological advances as AI in the field of plastic surgery. The authors are optimistic that, equipped with the basic tools contained herein, plastic surgeons will be able to navigate technical conversations with peers, trainees, patients, and technical partners for collaboration and to usher in a new era of technology in plastic surgery. Most importantly, they will be able to think critically about study design involving AI and plastic surgery. For now, we will continue to identify and explore opportunities related to AI that can benefit plastic surgeons and we encourage our peers to publish their work about AI as it relates to aesthetic surgery to raise awareness about the potential applications in our field.
